# TGF-β1 overexpression in severe COVID-19 survivors and its implications for early-phase fibrotic abnormalities and long-term functional impairment

**DOI:** 10.3389/fimmu.2024.1401015

**Published:** 2024-08-29

**Authors:** Enrique Alfaro, Raquel Casitas, Elena Díaz-García, Sara García-Tovar, Raúl Galera, María Torres-Vargas, María Fernández-Velilla, Cristina López-Fernández, José M. Añón, Manuel Quintana-Díaz, Francisco García-Río, Carolina Cubillos-Zapata

**Affiliations:** ^1^ Respiratory Diseases Group, Respiratory Service, La Paz University Hospital, IdiPAZ, Madrid, Spain; ^2^ Biomedical Research Networking Centre on Respiratory Diseases (CIBERES), Madrid, Spain; ^3^ Department of Intensive Medicine, La Paz University Hospital, Madrid, Spain; ^4^ Faculty of Medicine, Autonomous University of Madrid, Madrid, Spain

**Keywords:** post-COVID, acute respiratory distress syndrome, lung fibrosis, transforming growth factor, gas transfer components

## Abstract

**Introduction:**

In post-COVID survivors, transforming growth factor-beta-1 (TGF-β1) might mediate fibroblast activation, resulting in persistent fibrosis.

**Methods:**

In this study, 82 survivors of COVID-19-associated ARDS were examined at 6- and 24-months post-ICU discharge. At 6-months, quantitative CT analysis of lung attenuation was performed and active TGF-β1 was measured in blood and exhaled breath condensate (EBC).

**Results:**

At 6-months of ICU-discharge, patients with reduced DmCO/alveolar volume ratio exhibited higher plasma and EBC levels of active TGF-β1. Plasma TGF-β1 levels were elevated in dyspneic survivors and directly related to the high-attenuation lung volume. In vitro, plasma and EBC from survivors induced profibrotic changes in human primary fibroblasts in a TGF-β receptor-dependent manner. Finally, at 6-months, plasma and EBC active TGF-β1 levels discriminated patients who, 24-months post-ICU-discharge, developed gas exchange impairment.

**Discussion:**

TGF-β1 pathway plays a pivotal role in the early-phase fibrotic abnormalities in COVID-19-induced ARDS survivors, with significant implications for long-term functional impairment.

## 1 Introduction

Acute respiratory distress syndrome (ARDS) stands out as the most fatal manifestation of COVID-19, marked by the development of refractory respiratory failure and multiorgan dysfunction (1). However, the impact of ARDS extends beyond immediate mortality, profoundly affecting survivors (2, 3). The process of alveolar-capillary membrane permeabilization and repair, a critical aspect of ARDS recovery, often leaves survivors with persistent respiratory symptoms linked to fibrotic changes (4, 5).

Lung fibrosis emerges as a recognized sequel in post-COVID-19 patients, exerting significant repercussions on pulmonary function, such as gas exchange impairment and restrictive ventilatory disorder (6). Notably, these functional alterations as well as fibrotic-like lesions on computed tomography typically require some degree of evolution of fibrotic processes (7). In contrast, the gas transfer membrane component has been posited as a viable approach for the early detection of alterations in the alveolar-capillary membrane (8). A reduction in the alveolar-capillary membrane diffusing capacity for carbon monoxide (DmCO)/alveolar volume (VA) ratio suggests thickening of the alveolar-capillary membranes, concurrent with isotropic changes in alveolar dimensions (9–11). It is worth noting that a diminished DmCO/VA ratio exhibits high sensitivity for the early detection of lung interstitial disease, closely associated with incipient fibrotic alterations such as reticular opacities or honeycombing (12).

Beyond early detection, understanding the clinical impact of lung fibrosis secondary to ARDS caused by COVID-19 (13), underscores the importance of identifying the underlying mechanisms of the early-onset fibrosis pathway. During ARDS, matrix metalloproteinases, oxidative stress, inflammation, infiltrating leukocytes and macrophages, and cytotoxic mediators contribute to lung epithelial injury (14, 15). In some of the survivors of the acute phase, the imbalance between profibrotic and antifibrotic signals during the repair process may drive the development of an excessive fibroproliferative response (16). The regulation of this process could hinge on the key role played by transforming growth factor beta 1 (TGF-β1), a recognized mediator of tissue repair (17). In fact, TGF-β1 induces the expression of fibrotic mediators (18, 19) and has implications in the development of other fibrosing diseases, such as idiopathic pulmonary fibrosis (20) or systemic sclerosis (21).

We hypothesized that in certain survivors of ARDS secondary to COVID-19, the overexpression of TGF-β1, both in plasma and airways, may mediate fibroblast activation, prompting early alterations in the cellular repair process that may potentially result in long-term fibrosis. Consequently, 6-months after ICU-discharge from severe ARDS caused by COVID-19, we analyzed, as primary endpoint, the expression of active TGF-β1 and, as secondary endpoints, other fibrosis-related molecules in plasma and exhaled breath condensate (EBC) in relation to the presence or absence of alveolar-capillary membrane thickening, estimated through the DmCO/VA ratio. Additionally, an *in vitro* fibroblast model was examined to elucidate possible underlying mechanisms. Finally, we evaluated the discriminative capacity of TGF-β1 levels at 6-months to identify patients exhibiting a functional pattern of lung fibrosis at 24-months post-discharge.

## 2 Materials and methods

### 2.1 Study subjects

We enrolled 82 consecutive participants, aged 18 or older, who survived severe ARDS associated with COVID-19, meeting Berlin criteria and requiring invasive mechanical ventilation for at least 7 days and who were discharged from our hospital between February 2020 and January 2021. SARS-CoV-2 infection was confirmed by positive reverse-transcriptase polymerase chain reaction on nasal swab or tracheal aspirate at the time of ARDS. Patients were associated to COVID-19 initial Wuhan wave and were recruited six-months after ICU-discharge between September 2020 and July 2021 (**Supplementary Figure S1**). Detailed selection criteria can be found in the **Supplementary Methods** section. Written consent was obtained from all participants, and the study received approval from the institutional Ethics Committee at La Paz University Hospital (PI-4189).

Demographic, clinical, and ICU management data were recorded. At 6-months post-ICU discharge, anthropometric parameters, smoking status, comorbidities, and current treatment were documented. Respiratory symptoms were assessed using the European Community for Coal and Steel Questionnaire and the modified Medical Research Council dyspnea scale. Prior to lung function tests, a venous blood sample was drawn and EBC was sampled during 10 min of relaxed tidal breathing. Spirometry, lung volumes and diffusing capacity of the lungs for carbon monoxide (DLCO) measurements were conducted using MasterScreen (Viasys, CareFusion, Germany) according to current standardization. Lung diffusing capacity for nitric oxide was also measured following ERS recommendations (9). The membrane component of diffusing capacity (DmCO) and the pulmonary capillary blood volume (Vc) were calculated, and reference equations proposed by Zavorsky et al. were used (9). Lung morphology was assessed with a non-contrast chest CT scan with a quantitative analysis of lung attenuation using automated software.

Measurements of lung volumes and DLCO were repeated at 24-months after ICU discharge.

Detailed information on all clinical, functional and image measurements is available in the Online **Supplementary Data** and **Supplementary Figure 2**.

### 2.2 Plasma and peripheral blood mononuclear cells isolation

Blood obtained by venipuncture into ethylenediaminetetraacetic acid (EDTA) tubes was layered on top of 10 mL Ficoll-Paque Plus (Amersham Biosciences, Amersham, UK) and centrifuged for 20 minutes at 1500 rpm at room temperature. Plasma was removed from the upper layer; peripheral blood mononuclear cells (PBMCs) were removed from the interphase and washed in phosphate buffered saline for later RNA extraction.

### 2.3 Human primary fibroblasts cell culture

Human primary fibroblasts of healthy donors were obtained and expanded. Fibroblasts were cultured in Dulbecco’s modified eagle medium (DMEM) supplemented with 10% fetal bovine serum (FBS) at 37°C and 5% CO2, subsequent passing was performed using trypsin 0,02% and neutralized in equal volume of FBS. Fibroblasts on passage 8 were seeded in m24 plates (30.000 fibroblast/well), where they rested for 16 hours. Next, fibroblasts were treated with TGF-β1 signaling inhibitor, SB431542 (10µM) and after 3 hours they were stimulated with healthy volunteer or ARDS survivors’ plasma or EBC (10%) and cultured for 24 hours under those conditions. Fibroblasts supernatant was recovered, and cells were harvested for mRNA extraction or protein extraction. Protein extract was obtained by incubating cell pellet for 45 minutes in 200µL of specific lysis buffer from MARCKS ELISA kit (Raybiotech; Ref: PELMARCKS-S152-T) complemented with 1% protease inhibitor and 2% phosphatase inhibitor.

### 2.4 Soluble protein quantification

Protein concentration was determined in plasma, EBC and protein extracts using ELISA kits. Free active TGF-β1 isoform was assessed by using the LegendMax™ ELISA kit (BioLegend; ref. 437707; CA, USA), following the manufacturer’s instructions. Additionally, the following ELISA kits were employed following the manufacturer’s instructions: MMP2 (Invitrogen; ref. HC3081; MA, USA), α-SMA (FineTest; ref. EH1509; Wuhan, China), MARCKS (RayBiotech; ref. PELMARCKS-S152-T; GA, USA). EBC protein concentrations were quantified by ELISA as previously performed by other publications (22) and values were normalized to total protein concentration quantified by bicinchoninic acid (BCA) protein assay (Thermofisher Scientific; Ref. 23225; MA, USA). Additionally, α-SMA and phospho-MARCKS were quantified in fibroblasts’ protein extract following MARCKS ELISA kit instructions and reagents.

### 2.5 mRNA isolation and qPCR analysis

RNA was extracted from PBMCs or fibroblasts using High Pure RNA Isolation Kit (Roche Diagnostics, Switzerland). 0.25 μg of RNA was retrotranscribed using High-Capacity cDNA Reverse Transcription kit (Applied Biosystems; MA, USA). Real time quantitative PCR (RTqPCR) was performed using NZY Supreme qPCR Green MasterMix (Nzytech; Lisboa, Portugal) and specific primers for targeted genes synthesized by Eurofins Genomics Srl (Vimidrone, Italy) (**Supplementary Table S1**). RTqPCR was performed with CFX96 Touch Real-Time PCR Detection System (Bio-Rad Laboratories; CA, USA) for 40 cycles and results normalized to housekeeping gene 18S expression. Primer specific conditions for RTqPCR are specified in **Supplementary Table S2**.

### 2.6 Statistical analysis

Detailed information regarding statistical analysis and replicates can be found in figure legends. Generally, categorical variables are presented as numbers with percentages, and continuous variables as mean ± standard deviation or median (interquartile range) according to their distribution. Comparisons between subgroups were analyzed by Fisher exact test or Mann-Whitney test. For the *in vitro* model, group differences were analyzed by two-way ANOVA with Sidak’s multiple comparison test. The relationship between variables was assessed with Spearman correlation analysis. Due to multiple hypotheses testing, p-values have been adjusted by using Benjamini-Hochberg correction for false discovery rate.

To assess the discrimination ability of long-term impairment in total lung capacity (TLC) and DLCO, the area under the receiver operating characteristic curve (AUROC) was estimated and the Youden’s method was used to find an optimal cut-off point. Data were analyzed with GraphPad Prism v.8 and SPSS 25.0 software. Differences were considered statistically significant with P-values.

## 3 Results

### 3.1 Characteristics of study subjects

Detailed characteristics of survivors who experienced severe ARDS secondary to COVID-19 are outlined in **Table 1**. Additionally, **Supplementary Table S2** and **Supplementary Figure S2** provide a summary of symptomatic, functional, and morphological alterations observed at 6-months post-discharge, along with details regarding the ventilatory pattern during EBC collection. At this moment, 36 patients (43.9%) had a DmCO/VA ratio below the lower limit of normal. Further comparison between subgroups of patients with normal or reduced DmCO/VA ratio is also presented in both **Table 1**, **Supplementary Table S2**. Additionally, 21 patients (25.6%) exhibited reduced DLCO at 6-months post-discharge.

**Table 1:** General characteristics of the study subjects.

Characteristic	Overall group (n=82)	Subjects with normal DmCO/VA (n=46)	Subjects with decreased DmCO/VA (n=36)	p-Value
Males, n (%)	54 (65.9)	29 (63.0)	25 (69.4)	0.356
Age, years	59 ± 10	59 ± 11	59 ± 9	0.991
Body mass index, Kg/m^2^	30.5 ± 5.0	31.0 ± 5.0	29.8 ± 5.1	0.278
Fat mass index, Kg/m^2^	10.9 ± 4.7	11.4 ± 4.4	10.2 ± 5.0	0.252
Current or former smokers, n (%)	6 (11.1)	4 (14.8)	2 (7.4)	0.334
Main comorbidities prior to ICU admission, n (%)				
Hypertension	51 (62.2)	26 (56.5)	25 (69.4)	0.167
Dyslipidemia	40 (48.8)	20 (43.5)	20 (55.6)	0.194
Obesity	39 (47.6)	27 (58.7)	12 (33.3)	0.019
Diabetes	16 (19.5)	11 (23.9)	5 (13.9)	0.197
Cardiovascular diseases	13 (15.9)	7 (15.2)	6 (16.7)	0.547
Hypothyroidism	10 (12.2)	4 (8.7)	6 (16.7)	0.225
Respiratory diseases	8 (9.8)	4 (8.7)	4 (11.1)	0.498
Number of prior comorbidities	1 (1-1)	1 (0.5-1)	1 (1-1)	0.688
ICU admission parameters				
APACHE-II	15 ± 4	16 ± 4	14 ± 4	0.251
PaO_2_/FiO_2_	116 ± 55	114 ± 50	118 ± 63	0.747
Lymphocytes, x 10^3^/µl	747 ± 747	682 ± 369	830 ± 1051	0.425
D-dimer, ng/ml	1843 (1386-21924)	1772 (1411-20813)	8512 (1230-21924)	0.125
C-reactive protein, mg/L	162 ± 113	161 ± 110	163 ± 119	0.928
IL-6, pg/ml	772 (110-1000)	772 (274-1471)	577 (110-1000)	0.970
Invasive mechanical ventilation duration, days	16 (9-49)	16 (11-67)	21 (8-49)	0.706
Respiratory parameters on intubation				
Plateau pressure, cmH_2_O	26 ± 5	24 ± 2	29 ± 5	0.011
Peak inspiratory pressure, cmH_2_O	32 ± 5	31 ± 5	34 ± 5	0.229
Positive end-expiratory pressure, cmH_2_O	12 (12-13)	12 (10-14)	13 (12-13)	0.163
Driving pressure, cmH_2_O	11 (10-12)	11 (11-12)	11 (10-12)	0.451
Static compliance, ml/cmH_2_O	41 ± 14	42 ± 13	38 ± 16	0.591
Prone position, n (%)	65 (79.3)	39 (84.8)	26 (72.2)	0.132
Tracheostomy, n (%)	41 (60.3)	20 (54.1)	21 (67.7)	0.184
Extracorporeal membrane oxygenation, n (%)	3 (4.7)	1 (2.9)	2 (6.7)	0.452
Complications during ICU stay, n (%)				
Nosocomial infection	37 (80.4)	20 (80.0)	17 (81.0)	0.617
Pleural effusion	5 (7.7)	2 (5.7)	3 (10.0)	0.426
Pulmonary thromboembolism	23 (37.7)	12 (36.4)	11 (39.3)	0.511
ICU-acquired weakness	36 (90.0)	21 (91.3)	15 (88.2)	0.574
Hyperactive delirium	28 (73.7)	13 (65.0)	15 (83.3)	0.181
Reintubation	7 (10.9)	3 (8.6)	4 (13.8)	0.393
ICU readmission	5 (10.9)	2 (7.1)	3 (16.7)	0.294
Weight loss in ICU, Kg	5 (3-11)	5 (3-11)	5 (3-12)	0.970
Home oxygen therapy at discharge, n (%)	11 (16.4)	6 (15.8)	5 (17.2)	0.565
ICU length of stay, days	23 (11-46)	23 (12-40)	28 (11-56)	0.708
Hospital length of stay, days	45 (24-73)	42 (25-70)	53 (22-76)	0.527

Values are mean ± standard deviation, median (interquartile range) or number (percentage) according to their type and distribution. ICU, Intensive Care Unit; APACHE, Acute Physiology, Age, and Chronic Health Evaluation; DmCO, diffusing capacity of the membrane; PaO_2_/FiO_2_, ratio of arterial oxygen partial pressure to fractional inspired oxygen; IL-6, interleukin 6; VA, alveolar volume.

### 3.2 Active TGF-β1 in post-ARDS patients with early signs of alveolar-capillary membrane involvement

**Figure 1:**
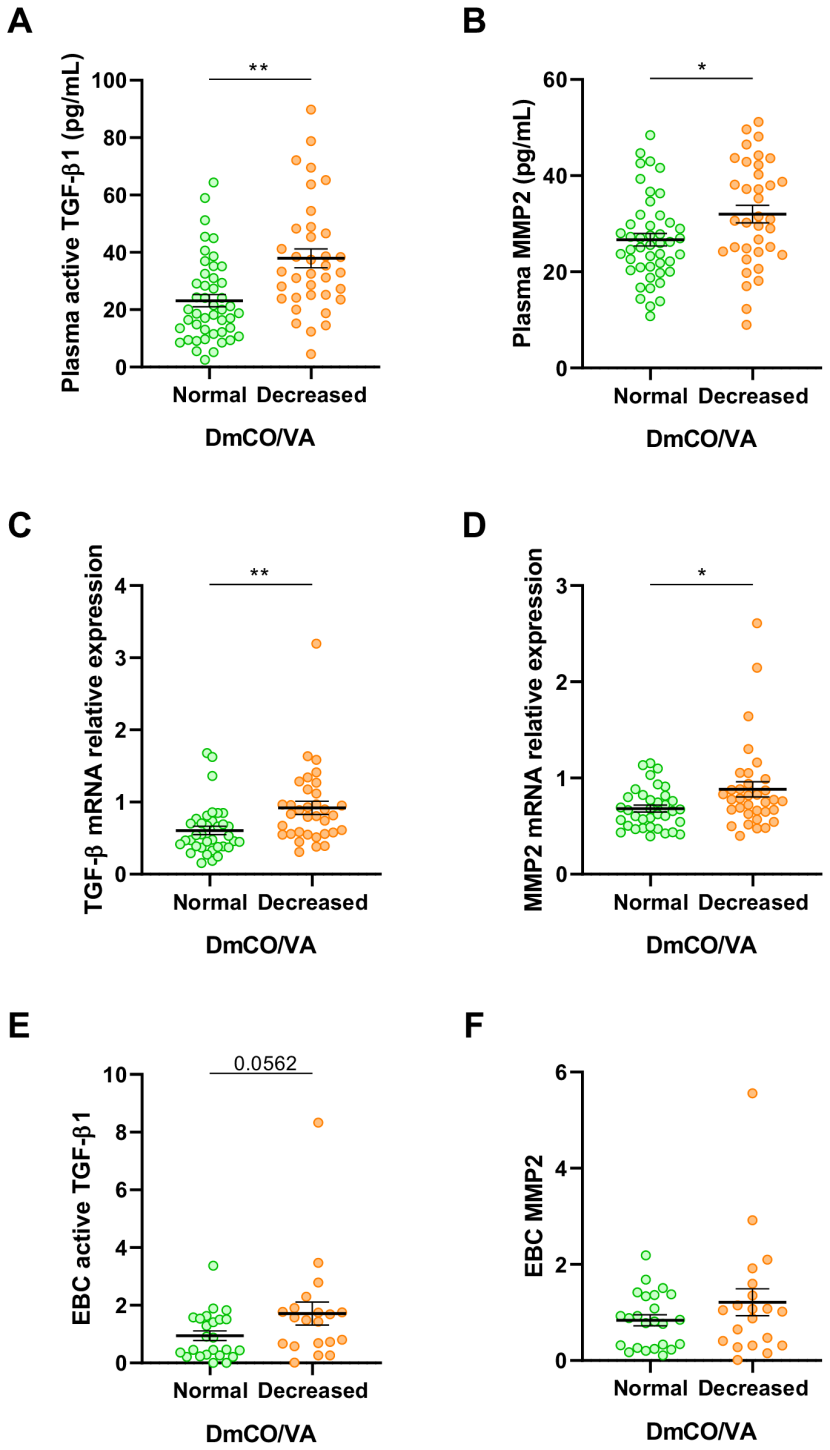
Elevated fibrotic markers in patients stratified by DmCO/VA. **(A)** Active TGF-β1 quantified by ELISA in plasma from patients with normal DmCO/VA (Green, N=46) and patients presenting reduced DmCO/VA (Orange, N=36). **(B)** MMP2 quantified by ELISA in plasma from patients with normal DmCO/VA (Green, N=46) and patients presenting reduced DmCO/VA (Orange, N=36). **(C)** mRNA expression of TGF-β quantified by RT-qPCR in PBMCs from patients with normal (Green, N=36) or reduced DmCO/VA (Orange, N=34). **(D)** mRNA expression of MMP2 quantified by RT-qPCR in PBMCs from patients with normal (Green, N=36) or reduced DmCO/VA (Orange, N=34). **(E)** Active TGF-β1 concentration normalized to total protein concentration in EBC from patients with normal (Green, N=24) or reduced DmCO/VA (Orange, N=20). **(F)** MMP2 concentration normalized to total protein concentration in EBC from patients with normal (Green, N=24) or reduced DmCO/VA (Orange, N=20). Data are represented as mean ± standard error of the mean. Differences between groups are analyzed by Mann-Whitney test and adjusted p-value is shown. *: adjusted-p<0.05; **: adjusted-p<0.01.

At 6-months of the ICU discharge, patients with reduced DmCO/VA exhibited higher plasma levels of active TGF-β1 isoform and matrix metalloproteinase 2 (MMP2) in comparison with those subjects with normal DmCO/VA (**Figures 1A, B**). As a complementary approach, we observed that the mRNA expression of TGF-β1 and MMP2 in PBMCs from patients with reduced DmCO/VA was also higher than in those with normal DmCO/VA ratio (**Figures 1C, D**). Furthermore, when stratifying ARDS survivors according to DmCO/VA ratio, those with a decreased ratio showed higher levels of active TGF-β1 in EBC than patients with normal DmCO/VA (**Figures 1E, F**). Altogether, these data suggest that post-ARDS patients with an early fibrotic stage characterized by a reduced DmCO/VA ratio exhibited higher levels of TGF-β1 in plasma and EBC compared to patients with preserved lung function.

### 3.3 Clinical and morphological implications of active TGF-β1 plasma levels in post-ARDS patients

**Figure 2:**
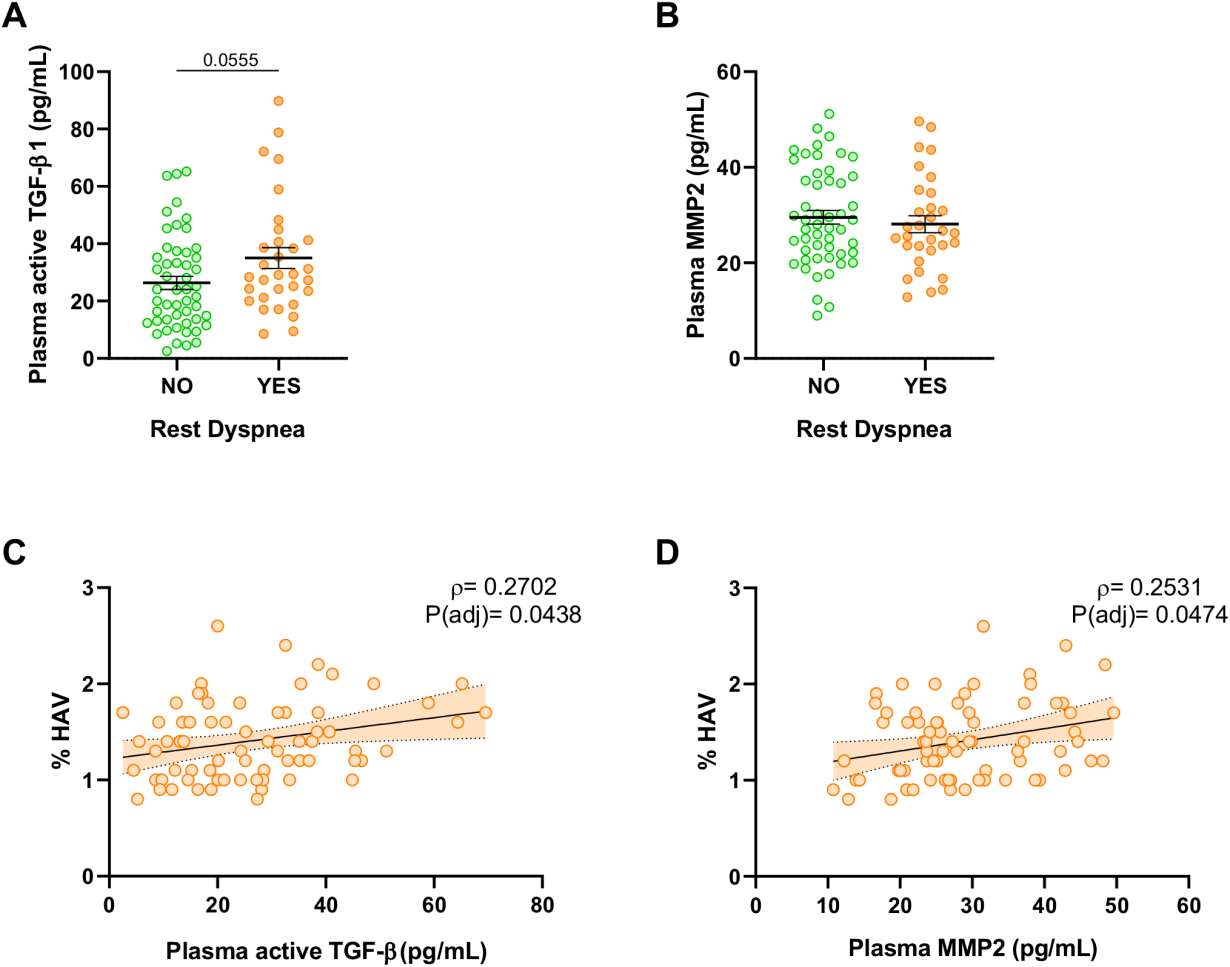
Plasma soluble fibrotic markers related to clinical characteristics of ARDS survivors. **(A)** Active TGF-β1 quantified by ELISA in plasma from patients without clinically relevant dyspnea (mMRC 0-1) (Green, N=51) and patients with dyspnea (mMRC ≥ 2) (Orange, N=31). **(B)** MMP2 quantified by ELISA in plasma from patients without clinically relevant dyspnea (mMRC 0-1) (Green, N=51) and patients with dyspnea (mMRC ≥ 2) (Orange, N=31). **(C, D)** Correlation of plasma active TGF-β1 and plasma MMP2 with the percentage of high-attenuation lung volume (HAV, %) in ARDS survivors (N=71). Data are represented as mean ± standard error of the mean. Differences between groups are analyzed by Mann-Whitney test and adjusted p-value is shown. Correlations are analyzed by Spearman’s method and Spearman’s Rho (ρ) and adjusted p-values [P(adj)] are shown. Correlation line estimates with 95% C.I. are represented.

Among survivors of ARDS secondary to COVID-19, individuals experiencing clinically relevant dyspnea at 6-months post-ICU discharge (mMRC ≥2) exhibited higher plasma TGF-β1 levels compared to the remained subjects (**Figure 2A**). However, no significant differences were observed in plasma levels of MMP2 (**Figure 2B**). Furthermore, in post-ARDS patients, the presence of elevated lung parenchymal attenuation, determined by high-attenuation lung volume (HAV), showed a direct proportional relationship with plasma levels of active TGF-β1 (**Figure 2C**) and MMP2 (**Figure 2D**, **Supplementary Table S3**). These findings underscore the potential clinical relevance of elevated plasma levels of active TGF-β1 in survivors of ARDS.

### 3.4 TGF-β1-mediated *in vitro* activation of human primary fibroblasts by ARDS survivors’ plasma and EBC

**Figure 3:**
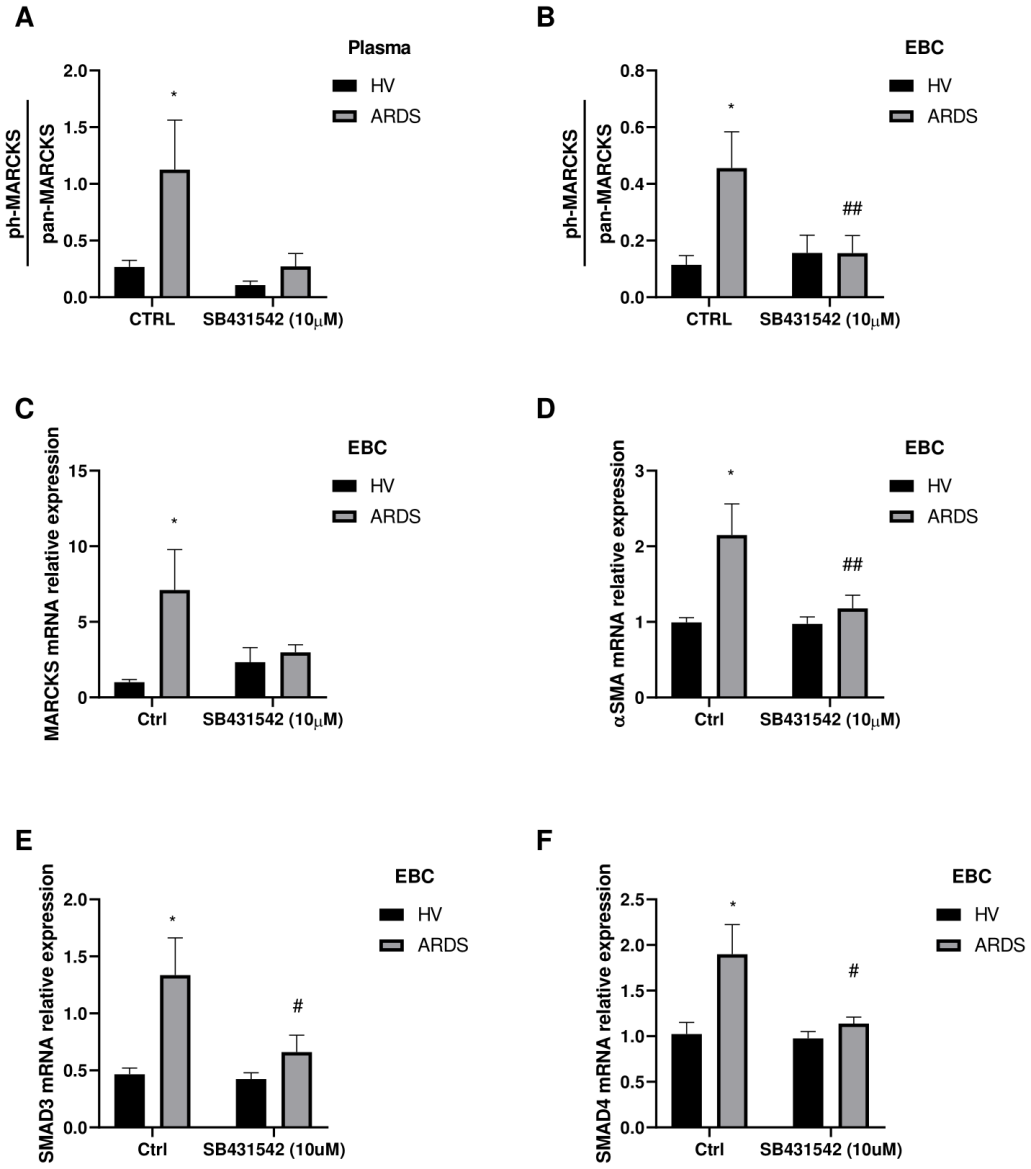
Fibroblasts’ activation *in vitro* model with plasma and EBC from ARDS survivors. Primary human fibroblasts are pre-treated with TGF-β receptor inhibitor (SB431542) 10µM for 3 hours or not (CTRL) and stimulated with healthy volunteer (HV) or ARDS survivors (ARDS) plasma or EBC and cultured for 24 hours. **(A)** Phosphorylated MARCKS (ph-MARCKS) to total MARCKS (pan-MARCKS) ratio measured in fibroblasts extracts treated with plasma from HV (N=6) or ARDS survivors (N=6). **(B)** Phosphorylated MARCKS (ph-MARCKS) to total MARCKS (pan-MARCKS) ratio measured in fibroblasts extracts treated with EBC from HV (N=5) or ARDS survivors (N=7). **(C–F)** mRNA expression of **(C)** MARCKS, **(D)** α-SMA, **(E)** SMAD3 and **(F)** SMAD4 measured in fibroblasts treated with EBC from HV (N=4) or ARDS survivors (N=5). Bars represent mean ± standard error mean. Differences are analyzed by 2-way ANOVA test and Sidak’s multiple comparison test. *: p-value <0.05 against HV-Ctrl; #: p-value<0.05, ##: p-value <0.01 against ARDS-Ctrl.

In order to explore the TGF-β1 role from plasma or EBC, we have performed an *in vitro* assay using primary human fibroblasts to analyze a specific marker of pulmonary fibrosis such as phosphorylated myristoylated alanine-rich C-kinase substrate (phosphor-MARCKS) (23). In particular, cells were cultured in presence of 10% of plasma or EBC from ARDS survivors or healthy volunteers for 24 hours. We observed an increase in phosphorylated MARCKS relative to total MARCKS in fibroblasts stimulated with ARDS survivors’ plasma (**Figure 3A**). However, when fibroblasts were pre-treated for 3 hours with 10µM SB431542 (TGF-β receptor inhibitor) (24, 25), the effect of ARDS survivors’ plasma was limited (**Figure 3A**). In addition, we performed similar experiment using patients’ or volunteers’ EBC instead of plasma as EBC presents more limited concentration of TGF-β1 and additionally may represent more accurately the pulmonary microenvironment. Fibroblasts treated with patients’ EBC also presented increased expression of phosphor-MARCKS compared to the ones treated with healthy volunteers’ EBC (**Figure 3B**). In accordance with previous plasma results, the effect of patients’ EBC was diminished when fibroblasts were pre-treated with SB431542 (**Figure 3B**). Moreover, we analyzed mRNA expression in fibroblasts stimulated with EBC and found that MARCKS and alpha smooth muscle actine (α-SMA) mRNA expression were higher in patients’ EBC-stimulated fibroblasts in a TGF-β receptor dependent manner (**Figures 3C, D**). In particular, α-SMA is a well-known and characterized protein used for assessment of activated fibroblasts in several tissues including the lung (26, 27). Collectively, our *in vitro* data suggested the capacity of patients’ plasma and EBC to promote profibrotic changes in human fibroblasts via TGF-β receptor.

### 3.5 Long-term consequences of plasma and EBC levels of TGF-β1 and fibrotic markers

As fibrotic lung lesions progress over the long term, patients experience gas exchange impairment and restrictive disorder. To explore these long-term alterations, we compared the levels of active TGF-β1 and other fibrotic markers 6-months after ICU discharge among patients who developed such conditions at 24-months after ICU discharge.

**Figure 4:**
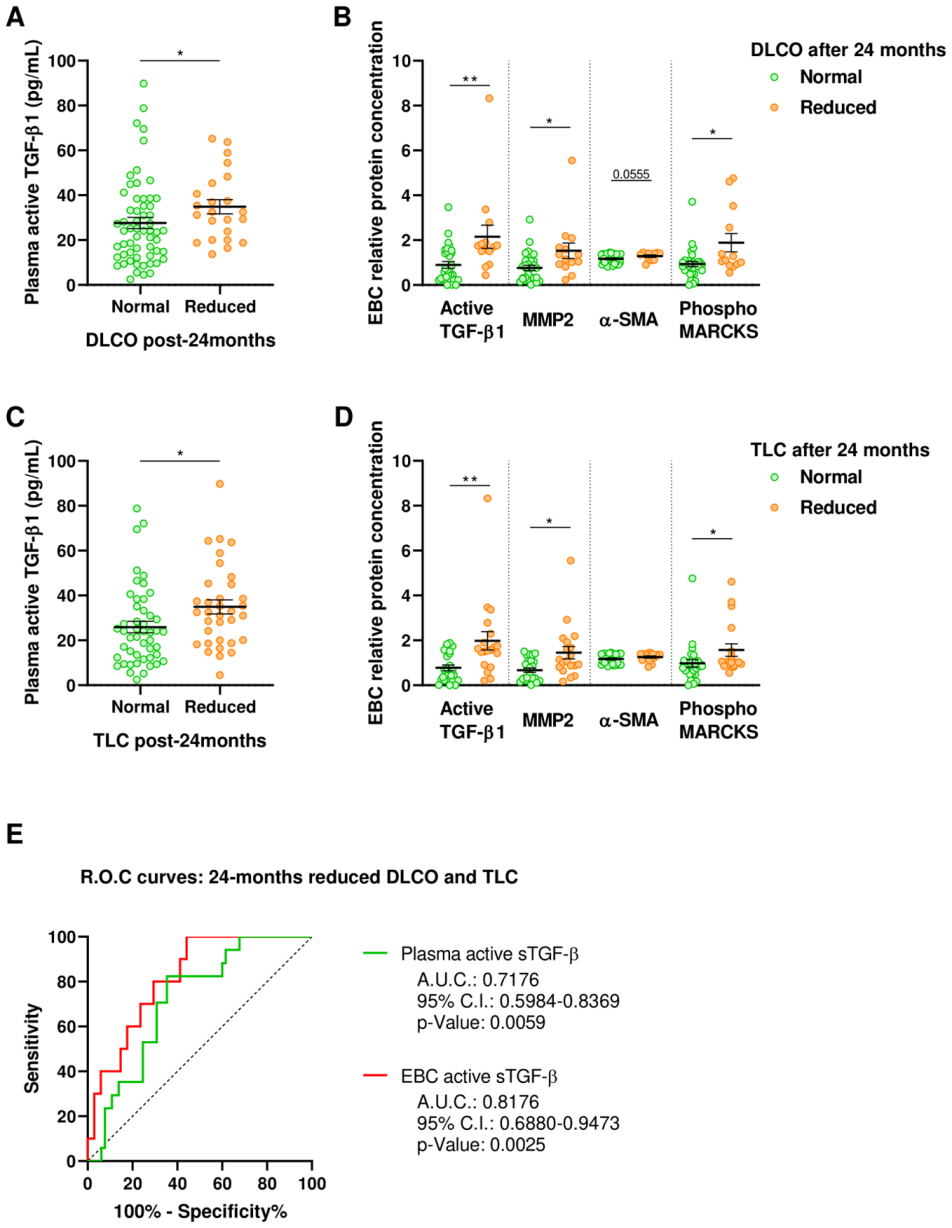
Fibrotic markers in ARDS survivors stratified by clinical characteristics 24-months post-ICU discharge. **(A)** Active TGF-β1 quantified by ELISA in plasma obtained 6-months post-ICU discharge from patients with normal (Green, N=59) or reduced DLCO (Orange, N=23) at 24-months post-ICU discharge. **(B)** Relative protein concentration of active TGF-β1, MMP2, α-SMA and phosphorylated MARCKS in EBC obtained 6-months post-ICU discharge from patients with normal (Green, N=30) or reduced DLCO (Orange, N=14) at 24-months post-ICU discharge. **(C)** Active TGF-β1 quantified by ELISA in plasma obtained 6-months post-ICU discharge from patients with normal (Green, N=48) or reduced TLC (Orange, N=34) at 24-months post-ICU discharge. **(D)** Relative protein concentration of active TGF-β1, MMP2, α-SMA and phosphorylated MARCKS in EBC obtained 6-months post-ICU discharge from patients with normal (Green, N=25) or reduced TLC (Orange, N=19) at 24-months post-ICU discharge. Data are represented as mean ± standard error of the mean. Differences between groups are analyzed by Mann-Whitney test and adjusted p-value is shown. *: adjusted-p<0.05; **: adjusted-p<0.01. **(E)** Receiver operating characteristic (ROC) curves for predictive performance value for 24-months reduced DLCO and TLC of plasma (green; N=82) and EBC active TGF-β1 (red; N=44). ROC curves were analyzed by Wilson/Brown test. Area under the curve (AUC), 95% confidence intervals (CI) and p-value are shown.

At 24-months post-ICU discharge, 23 patients (28.0%) with ARDS exhibited gas exchange impairment characterized by a reduced DLCO. Notably, patients with decreased DLCO at 24-months had higher plasma levels of active TGF-β1 at 6-months post-ICU discharge (**Figure 4A**). Furthermore, these patients also displayed elevated concentrations of active TGF-β1, MMP2, phosphor-MARCKS, and α-SMA in EBC at 6-months after ICU discharge (**Figure 4B**).

Similarly, 34 out of 82 ARDS survivors evaluated (41.5%) developed a restrictive disorder at 24-months post-ICU discharge, characterized by decreased total lung capacity (TLC). Compared to those maintaining normal TLC, these patients had higher plasma levels of active TGF-β1 at 6-months (**Figure 4C**) and elevated EBC concentrations of active TGF-β1, MMP2, and phosphor-MARCKS at 6-months post-ICU discharge (**Figure 4D**).

The plasma concentration of active TGF-β1 at 6-months post-ICU discharge discriminates patients who, at 24-months, experienced a reduction in both DLCO and TLC (AUROC: 0.718 ± 0.081, p=0.0059) (**Figure 4E**). Using the best-identified cut-off point (28.49pg/mL) yields a sensitivity of 82.4% (95% CI, 55.8-95.3%) and a specificity of 64.6% (95% CI, 51.7-75.8%). Moreover, the concentration of active TGF-β1 in EBC at 6-months post-ICU discharge demonstrates superior discriminative ability for identifying patients who developed long-term gas exchange impairment and restrictive disorders (AUROC: 0.818 ± 0.066, p=0.0025) (**Figure 4E**). The optimal cut-off point of 0.7673pg active TGF-β1/µg total protein results in a sensitivity of 100% (95% CI, 65.5-99.1%) and a specificity of 55.9% (95% CI, 38.1-72.4).

Taken together, our findings emphasize the relevance of fibrotic markers, especially TGF-β1, in ARDS patients. The medium-term levels of these markers appear to be associated with the progression of the fibrotic response, leading to both gas exchange impairment and restrictive disorders in the long term.

## 4 Discussion

In this study, we demonstrate that COVID-19 ARDS survivors who 6-months after ICU-discharge showed signs of alteration of the alveolar-capillary membrane, assessed by a reduction in the DmCO/VA ratio, have a higher expression of TGF-β1 active in both peripheral blood and respiratory tract. Notably, patients experiencing dyspnea display higher plasma levels of active TGF-β1, which correlate with less attenuation of the lung parenchyma. Additionally, using an *in vitro* model, we confirmed that plasma and EBC from survivors of COVID-19-ARDS can induce profibrotic changes in human primary fibroblasts. Furthermore, our findings reveal that levels of active TGF-β1 at 6-months can discriminate patients who, 24-months after discharge, will develop a restrictive disorder with gas exchange impairment.

Previously reported studies have highlighted a high incidence of fibrotic alterations in survivors of ARDS induced by COVID-19 (28). This fibrosis is likely influenced by various factors, including acute phase alterations of ARDS, lung lesions related to mechanical ventilation, maladaptive resolution of lesions, or an exaggerated tissue repair response (29). It is noteworthy that the development of pulmonary fibrosis necessitates the persistence of these alterations for an extended period. This is supported by the observation that the fibroproliferative response identified during ARDS hospitalization was associated with higher mortality but not with long-term fibrotic lung complications (30). Even before the COVID-19 pandemic, TGF-β1 was hypothesized to play a crucial role in maintaining the fibroproliferative process, with greater expression observed in bronchoalveolar lavage and lung biopsies of ARDS patients (31). Thus, the identification of an overexpression of active TGF-β1 in survivors of COVID-19-ARDS 6-months after ICU discharge underscores its potential role in post-COVID fibrosis, especially in those displaying early signs of alveolar-capillary membrane thickening.

TGF-β1, a member of a three-cytokine family expressed in various cells and tissues, is particularly important in lung epithelial cells and inflammatory cells (32). Unlike other isoforms, TGF-β1 has been associated with progressive pulmonary fibrosis (33). This cytokine, present in the extracellular matrix, requires activation through physical processes or mediation by specific integrins or proteases, including plasmin, thrombin, and matrix metalloproteinases 2 (MMP2) and MMP9 (34). Active TGF-β1 promotes profibrotic signals, facilitating the transition from fibroblasts to myofibroblasts and enhancing the accumulation of collagen, fibronectin, and other fibrosis-related extracellular matrix components (35, 36). Fatal COVID-19 cases are associated with increased TGF-β1 expression in the lung environment, resulting in disordered extracellular matrix assembly and prominent collagen deposition compared to other causes of ARDS (37). Moreover, the potential interaction between active plasma TGF-β1 and circulating immune cells should not be ignored. In this line, active TGF-β1 can exert anti-inflammatory and immune regulatory effect (38) however this is in stacking contrast with its effects on vascular endothelium. Active TGF-β1 can promote proinflammatory endothelial responses which may collaborate to vascular endothelium disruption (39). In post-COVID patients, endothelial and vascular lesions have been described, contributing to the influx of inflammatory immune cells into the lungs and potentially collaborating in profibrotic pathways (40, 41). Vascular endothelium disruption is known in ARDS, and if left unrepaired, it may contribute to the severity and persistence of lung injury (42, 43), supporting a loop of maladaptive injury and repair contributing to fibrosis.

Our *in vitro* model results further support the potential impact of TGF-β1-dependent profibrotic pathways in post-COVID-19 patients. They demonstrate that plasma and EBC from COVID-19 ARDS survivors can induce profibrotic alterations in healthy human fibroblasts. Additionally, this process increases the expression of components directly related to fibroblast remodeling, such as phosphorylated MARCKS and α-SMA (23, 26, 27). Notably, this effect is suppressed by blocking the TGF-β1 receptor. These data suggest an active extracellular matrix (ECM) remodeling process, potentially associated with a fibrotic lesion. They align with previous findings where bronchoalveolar lavage from survivors of ARDS of other origins induced fibroblast proliferation and α-SMA expression through the TGF-β1 receptor (31, 44, 45).

Finally, the ability of TGF-β1 levels 6-months after ICU discharge to discriminate patients who will develop functional alterations compatible with established pulmonary fibrosis 24-months later underscores the potential pathogenic role of this pathway. This raises questions about the utility of active TGF-β1 in plasma or EBC as a biomarker for future damage in survivors of COVID-19-ARDS. This is particularly important, as few risk markers for post-COVID-19 pulmonary fibrosis have been identified. Notably, recent reports suggest a relationship between fibrotic response in survivors of ARDS secondary to COVID-19 and exosomal down-regulation of miR-17-5p, miR-146a-3p, and miR-223-3p (46). Intriguingly, miR-17-5p downregulates the TGF-β receptor (47), while miR-223-3p alleviates TGF-β-induced epithelial-mesenchymal transition and extracellular matrix deposition (48). All of these findings reinforce the potential prognostic utility of active TGF-β1, enabling better patient selection for post-COVID pulmonary fibrosis treatment, including anti-fibrotic drugs.

Our study has several limitations, which we acknowledge. First, we lack information on premorbid clinical status, which makes it impossible to exclude preexisting conditions. Second, we were unable to access direct lung samples or BAL due to the invasiveness of these procedures. For the evaluation of the study biomarkers in the airways, we used exhaled breath condensate, which, although a less globally standardized procedure, has been previously employed in various respiratory diseases. Third, samples from the acute phase of ARDS were unavailable, except for commonly used clinical variables, preventing speculation on the timing of TGF-β1 pathway activation. Lastly, the local ethics committee deemed routine repetition of a CT scan unacceptable during the 24-month follow-up.

In conclusion, our study emphasizes the pivotal role of the TGF-β1 pathway in understanding early-phase fibrotic abnormalities in survivors of COVID-19-induced ARDS 6-months after ICU discharge. The analysis of EBC in these patients, representing a non-invasive approach to understanding the lung microenvironment, is noteworthy. Our *in vitro* model indicates that patients’ plasma and EBC can induce fibrotic changes in human fibroblasts through a TGF-β1 receptor-dependent pathway. Additionally, we propose a relationship between the TGF-β1 pathway measured 6-months after ICU discharge and lung function measured 24-months after discharge. The identification of soluble plasma markers contributing to the understanding and stratification of fibrotic complications and lung function impairment in COVID-19 ARDS survivors holds promise. Ultimately, understanding the pathways involved in the onset of pulmonary fibrosis in the context of COVID-19 ARDS survivors is crucial for developing therapeutic strategies that improve the quality of life and survival of these patients.

## Data Availability

The raw data supporting the conclusions of this article will be made available by the authors, without undue reservation.
