# Supplementary material for: TGF-β1 overexpression in severe COVID-19 survivors and its implications for early-phase fibrotic abnormalities and long-term functional impairment
Source: Front Immunol. 2024 Aug 29;15:1401015. doi: 10.3389/fimmu.2024.1401015 (PMC11393737; doi:10.3389/fimmu.2024.1401015)
Supplement: Supplementary file 1 [file DataSheet1.docx]

***Supplementary Material***

**TGF-β1 overexpression in severe COVID-19 survivors and its Implications for early-phase fibrotic abnormalities and long-term functional impairment**

**Enrique Alfaro^1,2,^ ^†^, Raquel Casitas^1,2, †^, Elena Díaz-García^1,2^, Sara García-Tovar^1^, Raúl Galera^1,2^, María Torres-Vargas^1,2^, María Fernández-Velilla^3^, Cristina López-Fernández^1,2^, José M Añón^3^, Manuel Quintana-Díaz^3, 4^, Francisco García-Río^1,2,4,*^, Carolina Cubillos-Zapata^1,2,*^**

**^†^First authorship:** These authors share first authorship

# Supplementary Methods

## Study subjects

We recruited 82 consecutive patients aged 18 years or older who had survived COVID-19-associated severe ARDS (defined according to the Berlin criteria) , requiring invasive mechanical ventilation for at least 7 days, with SARS-CoV-2 infection confirmed at that time by positive reverse-transcriptase polymerase chain reaction on nasal swab or tracheal aspirate. Exclusion criteria were persistent tracheostomy, need for permanent noninvasive ventilatory support, moderate-severe psychiatric conditions or cognitive deficits, hemodynamic instability, severe myopathy preventing ambulation, history of pulmonary resection, or respiratory infection in the 4 weeks prior to study testing. Workflow for patient screening and data collection is available as supplementary Figure S1.

All participants provided their written consent, and the study was approved by the institutional Ethics Committee (PI-4189).

## Data collection about severity of illness and course in the ICU

Data regarding demographic variables, PaO_2_/FiO_2_ ratio and APACHE II score at the time of ICU admission, mechanical ventilation parameters (plateau pressure, positive expiratory pressure, positive end-expiratory pressure, driving pressure, and static compliance), prone cycles required and the need for extracorporeal membrane oxygenation (ECMO) support, tracheostomy or reintubation were retrospectively registered from the patient’s electronic medical record. The treatment drugs (hypnotics, analgesics, muscle relaxants, corticoids and vasopressors), the complications developed (pressure ulcers, nosocomial infection, pleural effusion, deep venous thrombosis, pulmonary embolism, ischemic stroke, hyperactive delirium or ICU-acquired weakness), and ICU readmission were also recorded as well as the time of mechanical ventilation, duration of ICU stay, and duration of hospitalization.

## Clinical and functional assessment at 6 months after ICU discharge

Anthropometric characteristics were measured, including body composition (BF511 monitor, Omron Healthcare, Kyoto, Japan). Based on self-administered questionnaires and medical records, smoking status and comorbidities were recorded. Current smokers were defined as subjects who currently smoked more than 10 cigarettes per day. Past smokers who had quit smoking were not considered smokers in this study. All medications used by the participants at the time of the clinical evaluation were also listed.

The respective questions of the European Community for Coal and Steel (ECCS) Questionnaire were used to identify respiratory symptoms (cough, chronic expectoration, dyspnoea, wheezing or chest tightness). The degree of dyspnoea was evaluated by the modified Medical Research Council (mMRC) dyspnoea scale, that ranges between 0 to 4. It was considered that dyspnea was clinically relevant when it reached a grade equal to or higher than 2 on the mMRC scale.

Prior to lung function tests, a venous blood sample was drawn, and exhaled breath condensate (EBC) was sampled during 10 min of relaxed tidal breathing using an Ecoscreen condenser (Viasys, CareFusion, Würzbourg, Germany). Approximately 2 mL of condensate were collected and immediately stored at –80°C.

Spirometry, body plethysmography and measurement of diffusing capacity of the lungs for carbon monoxide (DLCO) were performed using a MasterScreen PFT system (Viasys) equipped with the SentrySuiteTM software, according to current standardization. Global Lung Initiative (GLI) equations were used as reference values and tests were interpreted according ERS/ATS technical strategies. Lung diffusing capacity for nitric oxide was also measured during a single breath maneuver according to the ERS recommendations.(1) After maximal expiration, the patients were requested to inhale fast as deeply in less than four seconds a gas mixture of 0.3% CO, 9.0% He, 21% O_2_ and 400 ppm NO in N2 and inhaled from a plastic bag containing a final concentration of NO of 50 ppm obtained ≤ 2 min before its use. The pressure curve displayed during the occlusion showed whether the patient has held his/her breath and the manoeuvre was accepted when pressure was <3kPa. A breath-hold of 5 s was then requested, followed by a rapid expiration. The first 0.75 L of expired gas were rejected and the following 0.75 L were sampled in a bag, which was automatically analysed for NO, CO and He. This washout volume was 0.5 L for subjects with a vital capacity < 2L. The actual breath-hold time was calculated using the Jones and Meade method.(2) The linearity of the electrochemical cell was checked by factory and the apparatus was calibrated for gas fractions using automated procedures. The procedure was repeated after a 4-min wait, and it was accepted if two successive DLNO and DLCO measurements were within 17 and 3 ml/min/mmHg, respectively. If this was not the case, additional measurements (up to five in total) were performed. The mean of two chosen manoeuvres was used for the subsequent analyses. The inert gas, He, was used in the calculation of alveolar volume (VA) by means of the He-dilution technique. The values of the membrane component of diffusing capacity for CO (Dm) and of the pulmonary capillary blood volume (Vc) were calculated according to the model by Guénard et al.(3) All diffusing capacity values were corrected for the haemoglobin levels obtained on the same day of the study and interpreted according to the reference equations proposed by Zavorsky et al.(1) Variables were expressed as a percentage of the predicted value and were considered reduced when they were below the lower limit of normal (LLN).

Measurements of lung volumes and DLCO were repeated 24 months after ICU discharge.

## Lung image analysis

CT scans were obtained in the supine position at full inspiration with a 320-row multidetector CT scanner (Aquilion ONE, Canon Medical Systems, Japan) with the following specific settings: exposure parameters: 100 Kv and automatic exposure control; scan parameters: cranio-caudal scan with 80x0.5 mm collimation, pitch 0.8 and 0.35s rotation time, reconstruction: 0.5 mm slice with 0.4 interval.

A quantitative study of the lung parenchyma (volume and attenuation) was performed using a validated automated CT processing software (CT Pulmo 3D, SyngoVia® VB60A, Siemens Healthineers, Germany). Mean lung density (MLD) in Hounsfield units (HU), high attenuation value (HAV) as percentage of lung tissue with HU above -700, and 15^th^ percentile of HU were calculated

# Supplementary Figures and Tables

## Supplementary Tables

**Table S1.** Primer sequences and specific conditions used in qPCRs in this study which were provided by Eurofins Genomics.

| Target | Primer | Sequence | Annealing temperature (time=10sec) | Extension time (temperature =70ºC) | Reading temperature |
| --- | --- | --- | --- | --- | --- |
| TGF-β | Forward | CTAATGGTGGAAACCCACAACG | 62ºC | 60sec | 88ºC |
|  | Reverse | TATCGCCAGGAATTGTTGCTG |  |  |  |
| MMP2 | Forward | GATACCCCTTTGACGGTAAGGA | 61ºC | 22sec | 82ºC |
|  | Reverse | CCTTCTCCCAAGGTCCATAGC |  |  |  |
| α-SMA | Forward | CTGACTGAGCGTGGCTATTC | 55ºC | 90sec | 76ºC |
|  | Reverse | CCACCGATCCAGACAGAGTA |  |  |  |
| MARCKS | Forward | AGCCCGGTAGAGAAGGAGG | 57ºC | 90sec | 84ºC |
|  | Reverse | TTGGGCGAAGAAGTCGAGGA |  |  |  |
| SMAD3 | Forward | AACGGCCAGGAGGAGAAATG | 60ºC | 50sec | 86ºC |
|  | Reverse | ATCCAGGGACCTGGGGAT |  |  |  |
| SMAD4 | Forward | TGCATTCCAGCCTCCCATTT | 56ºC | 20sec | 75ºC |
|  | Reverse | CTCTCCTACCTGAACGTCCATT |  |  |  |
| 18S | Forward | CGGCGACGACCCATTCGAAC | 54ºC | 45sec | 80ºC |
|  | Reverse | GAATCGAACCCTGATTCCCCGTC |  |  |  |

| **Characteristic** | | **Overall group**  (n=82) | **Subjects with normal DmCO/VA**  (n=46) | **Subjects with decreased DmCO/VA**  (n=36) | **p-Value** |
| --- | --- | --- | --- | --- | --- |
| Respiratory symptoms, n (%) | | 62 (75.6) | 33 (71.7) | 29 (80.6) | 0.255 |
| Dyspnoea, n (%) | | 31 (37.8) | 14 (30.4) | 17 (47.2) | 0.092 |
| mMRC scale dyspnoea level | | 1 (0-2) | 1 (0-1) | 1 (0-2) | 0.138 |
| Lung function testing | | | | | |
|  | FVC, % pred. | 91 (79-100) | 90 (81-100) | 92 (79-98) | 0.834 |
|  | FEV_1_, % pred. | 89+16 | 94+15 | 95+13 | 0.615 |
|  | TLC, % pred. | 76+14 | 76+15 | 77+13 | 0.598 |
|  | FRC, % pred. | 63 (56-77) | 61 (51-73) | 67 (59-80) | 0.081 |
|  | DLCO, % pred. | 91 (75-106) | 92 (75-107) | 87 (75-104) | 0.379 |
|  | DLCO/VA, % pred. | 103 (92-116) | 108 (95-124) | 100 (91-111) | 0.086 |
|  | DmCO/VA, % pred. | 73 (57-128) | 119 (82-136) | 56 (45-67) | 0.019 |
|  | Vc/VA, % pred. | 140 (115-169) | 144 (119-176) | 129 (112-156) | 0.248 |
| Automated CT measurements of lung density | | | | | |
|  | Mean lung density, HU | -794  (-816 - -761) | -784  (-816 - -757) | -799  (-817 - -766) | 0.481 |
|  | Hight attenuation volume, % | 1.3 (1.1-1.7) | 1.4 (1.0-1.6) | 1.2 (1.1-1.7) | 0.638 |
|  | 15^th^ percentile, HU | -877  (-895 - -855) | -875  (-894 - -851) | -883  (-894 - -862) | 0.628 |
| Conditions of exhaled breath condensate (EBC) collection | | | | | |
|  | Time, min | 10 (10-12) | 10 (10-13) | 10 (10-11) | 0.560 |
|  | Tidal volume, l | 0.56  (0.41-0.78) | 0.53  (0.42-0.71) | 0.61  (0.41-0.91) | 0.371 |
|  | Cumulative ventilation, l | 110 (101-139) | 106 (101-133) | 113 (101-156) | 0.183 |
|  | Breathing frequency, min^-1^ | 19 (15-23) | 20 (15-24) | 18 (15-21) | 0.167 |

**Table S2**. Characteristics of the study subjects at 6 months of ICU discharge

Values are mean ± standard deviation, median (interquartile range) or number (percentage) according to their type and distribution.

Abbreviations: DLCO, diffusing capacity of the lungs for carbon monoxide; DmCO, membrane diffusing capacity; FEV_1_, forced expiratory volume at 1 second; FRC, functional residual capacity; FVC, forced vital capacity; HU, Hounsfield units; ICU, Intensive Care Unit; mMRC, modified Medical Research Council; TLC, total lung capacity; VA, alveolar volume; Vc, capillary blood volume.

**Table S3**. Relationship between plasma levels of TGF-ß and MMP2 with CT parameters of lung parenchyma attenuation

|  | Plasma active TGF-ß1 (pg/ml) | Plasma MMP2 (pg/ml) |
| --- | --- | --- |
| HAV (%) | ρ = 0.270, p=0.023 | ρ = 0.253, p=0.033 |
| MLD (HU) | ρ = 0.017, p=0.882 | ρ = -0.012, p=0.921 |
| 15th percentile (HU) | ρ = -0.007, p=0.949 | ρ = 0.010, p=0.928 |

Values are Spearman correlation coefficient (ρ) and p-value.

Abbreviations: TGF-ß1, transforming growth factor-beta-1; MMP2, matrix metalloproteinase 2; HAV, hight attenuation volume; MLD, mean lung density; HU, Hounsfield units

## Supplementary Figures


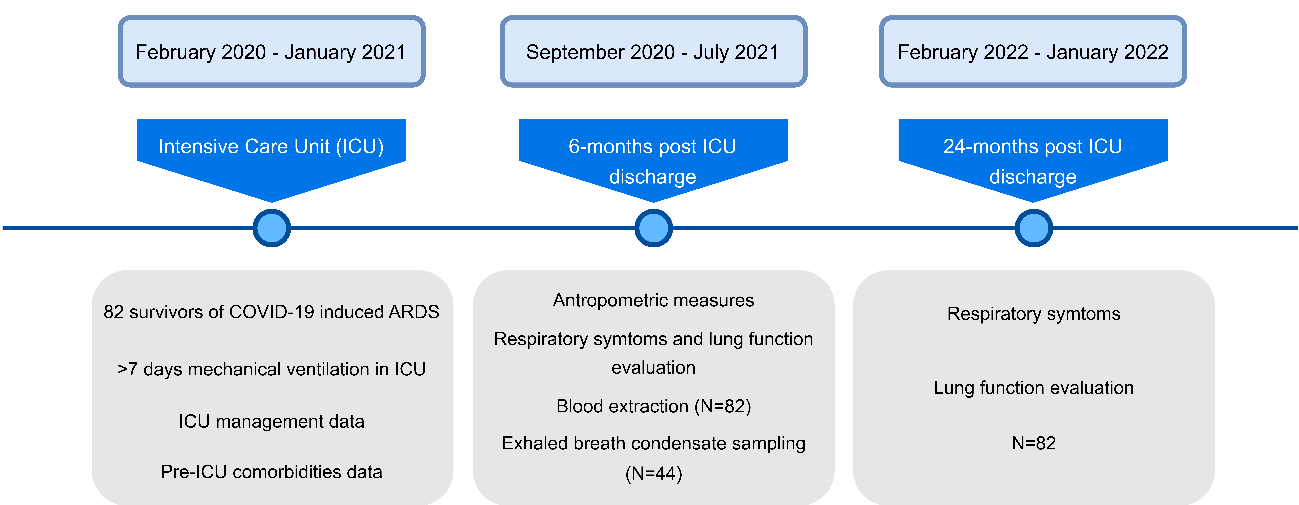
**Figure S1**. Comprehensive workflow for patient screening and data and sample collection.


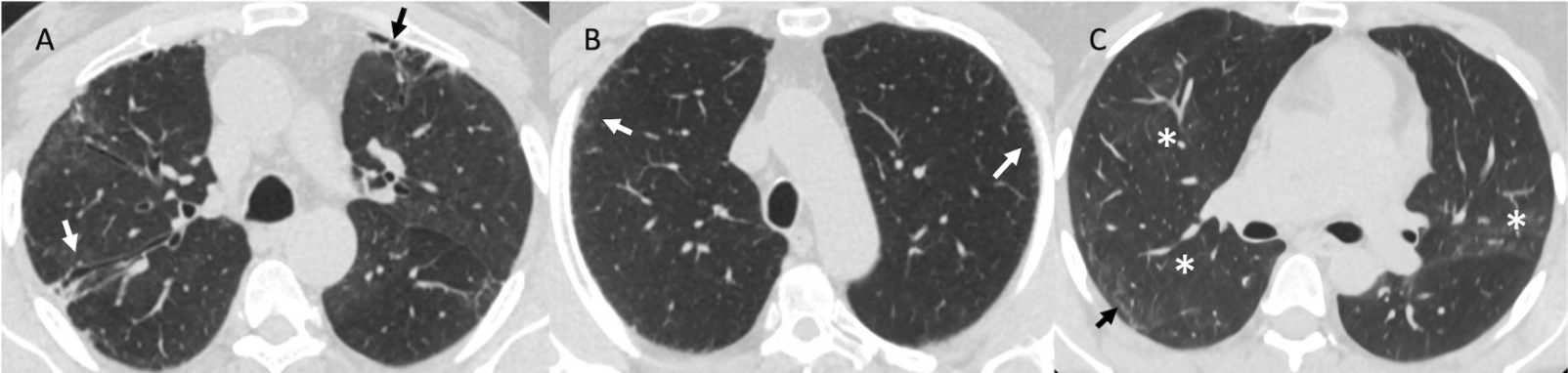


**Figure S2**. Examples of main CT patterns of post COVID-19 abnormalities: A) Unequivocal signs of fibrosis (left upper lobe volume loss, traction bronchiectasis [white arrow], and honeycombing [black arrow]). B) fibrotic-like abnormalities (fine linear and reticular opacities [white arrow]). C) non-fibrotic abnormalities (ground-glass opacity [*] and band-like opacities [black arrow]).

# Supplementary references

1. Zavorsky GS, Hsia CC, Hughes JM, Borland CD, Guénard H, van der Lee I, et al. Standardisation and Application of the Single-Breath Determination of Nitric Oxide Uptake in the Lung. *Eur Respir J* (2017) 49(2). Epub 20170208. doi: 10.1183/13993003.00962-2016.

2. Jones RS, Meade F. A Theoretical and Experimental Analysis of Anomalies in the Estimation of Pulmonary Diffusing Capacity by the Single Breath Method. *Q J Exp Physiol Cogn Med Sci* (1961) 46:131-43. doi: 10.1113/expphysiol.1961.sp001525.

3. Guénard HJ, Martinot JB, Martin S, Maury B, Lalande S, Kays C. In Vivo Estimates of No and Co Conductance for Haemoglobin and for Lung Transfer in Humans. *Respir Physiol Neurobiol* (2016) 228:1-8. Epub 20160305. doi: 10.1016/j.resp.2016.03.003.
